# Characterizing Host microRNA: Virus Interactions of *Orthoavulavirus javaense*

**DOI:** 10.3390/v16111748

**Published:** 2024-11-07

**Authors:** Megan C. Mears, Abhijeet Bakre

**Affiliations:** Exotic and Emerging Avian Viral Disease Research Unit, Southeast Poultry Research Laboratory, US National Poultry Research Center, 934 College Station Road, Athens, GA 30605, USA; megan.mears@usda.gov

**Keywords:** *Orthoavulavirus javaense*, Newcastle disease virus, miRNAs, viral seed mimic, viral seed sponge

## Abstract

Post-transcriptional gene regulation mediated by microRNAs (miRNAs) relies on sequence complementarity between the miRNA seed site and the target gene transcript(s). This complementarity can completely inhibit or reduce translation into protein. We hypothesized that viruses employ sequence complementarity/similarity with host miRNAs to inhibit or increase the miRNA-mediated regulation of host gene expression specifically during viral infection(s). In this study, we focus on *Orthoavulavirus javaense* (OAVJ), the causative of Newcastle disease, a poultry disease with significant economic impact. A computational analysis of OAVJ genomes from low-virulence (lentogenic) versus virulent (velogenic) viruses was carried out to identify viral signature motifs that potentially either mimic or complement host miRNA seed sequences. Data show that OAVJ genomes harbor viral seed mimics (vSMs) or viral seed sponges (vSSs) and can mimic host miRNAs or inhibit their regulation of host genes, disrupting cellular pathways. Our analyses showed that velogens encode a statistically significant higher number of vSMs and a lower number of vSSs relative to lentogens. The number of vSMs or vSSs did not correlate with gene length. The analysis of the secondary structures flanking these vSMs and vSSs showed structural features common to miRNA precursors. The inhibition or upregulation of vSS-miR-27b-5p altered P gene expression in a sequence-dependent manner. These data demonstrate that viral transcripts can interact with host miRNAs to alter the outcomes of infection.

## 1. Introduction

MicroRNAs (miRNA) are critical endogenous post-transcriptional regulators of gene expression in vertebrates and regulate nearly 60% of the vertebrate transcriptome [1]. MiRNA precursors are single-stranded RNAs that are processed into 18–25 nt long mature double-stranded RNAs; miRNA genes are also highly conserved across the evolutionary tree [2]. MiRNAs have important roles in regular cellular physiology and in regulating the immune and inflammatory response to infection or cellular stress [3,4,5,6,7,8].

Intracellular pathogens like viruses trigger the expression of host cytokines and interferons and lead to the establishment of an anti-viral state by inducing the expression of interferon-stimulated genes (ISGs). In addition, viral infections have been demonstrated to dysregulate host miRNA expression [9,10,11,12,13,14]. This dysregulation leads to the selective disruption of co-expressed genes and pathways and paves the way for the suppression of host immune responses and the establishment of infection or clearing of infection. Host cells have also developed a network of complementary endogenous RNAs (ceRNAs) to avoid the dysregulation of cellular signaling and consequent pathology [15,16,17,18,19,20,21,22,23].

Host miRNA biogenesis has been reviewed extensively and involves the nuclear processing of miRNA genes and the export of a pre-miRNA construct that undergoes further cytosolic processing, leading to the mature miRNA duplex [24,25]. The key aspect of miRNA-mediated post-transcriptional regulation is the interaction between nucleotides 2–8 at the 5′ end of an miRNA guide strand (the “seed” region) with the complementary sequence in its target transcript(s), ultimately leading to translation blocking or transcript degradation, leading to gene silencing [26]. Because of this short region of complementarity, a typical miRNA is predicted to target several hundred mRNAs; however, the real target repertoire may be restricted either due to tissue-specific gene expression, the lack of co-transcription of an miRNA and its target, or incorrect molecular stoichiometry [1]. The seed region is critical for miRNA function, as mutations in the seed region can abrogate miRNA targeting and function [27].

Many mammalian viruses encode their own miRNAs [28,29,30]. The major DNA viruses that infect poultry—Marek’s disease virus [31,32,33,34,35,36], Turkey herpesvirus [37,38], and infectious laryngotracheitis virus [37,39]—also encode their own miRNAs, and these play roles in regulating both host and viral translation. To date, RNA viruses have not been shown to encode miRNAs; however, we previously showed that RNA viruses encode sequence motifs that may modulate miRNA activity, either by mimicking miRNA seed sites referred to as viral seed mimics (vSMs), or, alternatively, viral genome/transcripts may block host miRNA activity by sponging their seed sites through sequence complementarity, referred to as viral seed sponges (vSSs) [40].

Newcastle disease (ND) is a major economic challenge for the poultry industry. ND outbreaks affect both wild birds and poultry, and are a persistent problem in South America, Asia, and parts of Europe [41]. *Orthoavulavirus javaense* (OAVJ) (formerly known as *Avian orthoavulavirus 1* (AOAV-1) or Avian paramyxovirus 1 (APMV-1), or Newcastle disease virus (NDV)) is the causative agent of ND and occurs worldwide. OAVJ is an enveloped paramyxovirus with a ~15kb negative-sense single-stranded RNA genome that encodes six genes: nucleocapsid (N), phosphoprotein (P), matrix (M), hemagglutinin (HN), fusion (F), and polymerase (L). Like other paramyxoviruses, OAVJ gene transcript abundance decreases from the 3′ to 5′ end [42,43]. All OAVJs can be classified genetically into two main classes: class I and class II. Class II viruses cause the majority of ND outbreaks and are further classified into 21 genotypes, of which genotypes V, VI, VII, and XIII are the most commonly circulating [44].

OAVJ strains are classified as either low virulence (lentogenic) or virulent (mesogenic/velogenic) [45]. High-virulence strains contain a polybasic cleavage site (^112^R/G/K-R-Q/K-K/R-R-F^117^) in the fusion (F) protein, in contrast to a monobasic cleavage site (^112^G-R/K-Q-G-R-L^117^) in low-virulence strains [46]. OAVJ viruses employ multiple different mechanisms to modulate the host immune response. These include avoiding detection by host innate immune responses, inhibiting intracellular signaling, including Jak-STAT pathways, modulating the expression of interferons and interferon-stimulated genes during infection, and altering the cell cycle [47,48,49]. Additional interferon signaling pathways can be inhibited by two proteins, V and W, which are generated from alternative splicing of the OAVJ P protein [50,51,52,53,54].

In this manuscript, we explore the possibility that OAVJ viruses have exploited the error-prone nature of RNA-dependent RNA polymerases to inundate the host with nonsense transcripts that interfere with the host’s recognition of infection. We hypothesize that one of the mechanisms by which OAVJ viruses interfere in the regulation of normal homeostasis is by modulating the host’s miRNA function and networks. Encoding miRNA-like seed mimics enables OAVJ viruses to impersonate host miRNAs (as vSMs) and dysregulate normal post-transcriptional gene regulation. Alternatively, vSS sequence motifs can potentially sequester host miRNAs away from their intended targets and derepress pro-viral gene networks or suppress anti-viral pathways. To determine vSM and vSS motifs in lentogenic, mesogenic, or velogenic OAVJs, genomic sequences were computationally analyzed and the potential for RNAse III processing was studied by secondary structure predictions of flanking regions. Finally, the impact of inhibiting or upregulating a predicted vSS, mir-27b-5p, on viral replication and gene expression was studied.

## 2. Materials and Methods

### 2.1. Sequence Analysis

Full-length genome nucleotide sequences for different pathotypes of OAVJ were downloaded from the National Center for Biotechnology Information (NCBI). We obtained 16 lentogenic, 5 mesogenic, and 78 velogenic OAVJ strain sequences, which were used for further analysis (accession numbers are listed in Supplementary Table S1). Mature chicken miRNA sequences (ver. 24) were downloaded from miRbase [55] and used to construct a local BLAST database in Galaxy server (ver. 23.0.3.dev0) [56] on the Ceres SciNet computing cluster. OAVJ genome sequences were analyzed for homology with chicken miRNAs using BLASTN-SHORT, with match/mismatch scores set to +1/−3, gap open/extend cost set to 3, and the expect value (E-value) set to 100. In BLASTN-SHORT, these match/mismatch scores of +1/−3 correspond to 99% identity between sequences [57]. The highest scoring pairs (HSPs) were exported to a csv hit table and then filtered and analyzed in MS Excel. Further, only HSPs with ≥90% identity with an alignment length of ≥11 nucleotides and covering the miRNA seed site (nucleotides 2–8) were considered “true” hits; this enabled the removal of non-seed matches. HSPs in the same 5′-3′ orientation as the mature miRNA (plus/plus orientation) were designated as viral seed mimics (vSMs), while those in the opposite orientation (plus/minus) were designated viral seed sponges (vSSs). The genomic coordinates of vSMs and vSSs were mapped to gene annotations on the query virus sequences manually after extracting feature metadata from the query Genbank files using Geneious ver 9.2. Data summaries were obtained using Pivot tables.

### 2.2. Viruses, Cell Culture, and Transfection Conditions

Two strains of OAVJ, the vaccine strain LaSota, and the velogenic strain Chicken/California/18-016505-1/2018 (genotype Vb) (CA18) were used in this study. Viruses were grown in the allantoic cavity of 9–10-day-old embryonated chicken eggs (ECEs) from SPF chickens [58].

Mycoplasma-free chicken fibroblast cells (DF-1) (CRL-3586, American Type Culture Collection (ATCC), VA, USA) were grown in DMEM containing high glucose, 1× antibiotic–antimycotic mix, 1 mM sodium pyruvate, 1× Glutamax, and 10% heat-inactivated fetal bovine serum (FBS) at 39 °C with 5% CO_2_, as per the conditions recommended by ATCC.

For transfections, DF-1 cells were plated at a density of 12–15,000 cells per well in a 96-well plate; the outermost rows and columns contained plain media only. Transfections were carried out with Dharmafect 1 (Horizon Discovery, Cambridge, UK) using the manufacturer’s recommended conditions. miRNA controls, inhibitor(s), or mimics (Dharmacon, part of ThermoFisher Scientific, Waltham, MA, USA) were transfected at final concentration of 25 nm. Infection followed by transfection experiments were carried out using either LaSota or CA18 viruses and by infecting DF-1 cells using a multiplicity of infection (MOI) of 5.

Cytotoxicity was measured using a Cell Titer Blue assay (Promega, Madison, WI, USA), as per the manufacturer’s recommendations. Briefly, media in wells were replaced with fresh media containing Cell Titer Blue (CTB) at a 10% volume, then incubated for 1.5 h, and absorbance/fluorescence was measured on a Synergy HTX plate reader (Biotek, Shoreline, WA, USA) using the manufacturer’s recommended settings. Wells with no cells or completely reduced media with CTB were used as negative and positive controls. Reductions in absorbance/fluorescence were plotted relative to controls to calculate percent cytotoxicity.

### 2.3. Secondary Structure Analysis

The secondary structure of the seed sponge/miRNA hybrid was obtained using the RNAhybrid [59] server. Whole P gene alignments or sequences containing a 100 nt 5′ and 3′ flank around the vSS target site were extracted from the P sequence alignments. Mature miRNA sequences were obtained from miRbase ver.24 [55].

### 2.4. qRT-PCR

Total RNA from transfected and/or infected cells was isolated using RNAzol RT (MRCgene, Cincinnati, OH, USA) using the manufacturer’s recommended conditions, but including 1–2 µL of Glycoblue during precipitation for easy pellet visualization. RNA was quantified using a Nanodrop 2000 spectrophotometer (ThermoFisher Scientific, USA). First-strand synthesis was carried out on equal amounts of RNA across all samples using an miRNA cDNA synthesis kit (Agilent, Santa Clara, CA, USA), as per the manufacturer’s recommendations. Briefly, equal amounts of total RNA were polyadenylated with *E. coli* Poly A polymerase at 37 °C for 30 min. Polyadenylated RNA was annealed to an adaptor oligo at 25 °C for 15 min and reverse-transcribed at 42 °C for 60 min, followed by the inactivation of reverse transcriptase at 95 °C for 5 min. First-strand cDNA was diluted 1:10 in molecular-grade water and used as a template for qPCR.

All qRT-PCR reactions contained 1 µL of 1:10 diluted cDNA, 2× Lunascript master mix (New England Biolabs (NEB), Ipswich, MA, USA), and gene-specific forward and reverse oligos (250 nM final concentration). Amplifications were carried out on a Quantstudio 5 (Applied Biosystems, Waltham, MA, USA) using the following program: 95 °C for 1 min, 40 cycles of 95 °C for 15 s, annealing at 56 °C for 30 s, and amplification at 60 °C for 30 s, followed by melt curve analysis. Data were analyzed using the ΔΔCt methodology [60], using GAPDH or 5.8 S rRNA (for miRNAs) as housekeeping genes. The forward and reverse primer sequences used for P were 5′-ATGGCCACCTTTACAGATG-3′ and 5′-CCGTGCTTCTCTCGTGCT-3′, respectively. PCRs for the M gene were performed using forward primer M+4100 5′-AGTGATGTGCTCGGACCTTC-3, probe M+4196 5′-[FAM]TTCTCTAGCAGTGGGACAGCCTGC[TAMRA]-3′, and reverse primer 5′-CCTGAGGAGAGGCATTTGCTA-3′as previously described [61].

### 2.5. Statistical Analysis

All statistical analyses were performed using GraphPad Prism ver. 10.2.3 (build 403). Statistical analyses were carried out using one-way ANOVA with repeated measures using Geisser–Greenhouse correction and either Tukey or Holm–Sidak post hoc testing for multiple comparisons. Data represent the mean ± the standard deviation of biological triplicates.

## 3. Results

### 3.1. Computational Analysis Suggests That OAVJ Genomes Encode Host miRNA Seed Mimics and Sponge Motifs

We hypothesized that OAVJ genomes can encode vSM/vSS motifs that can mimic or inhibit the activity of chicken miRNAs. To identify sequence motifs in OAVJ genomes that could function as vSMs or vSSs, we undertook a homology search between known lentogenic (n = 16), mesogenic (n = 5), and velogenic (n = 78) OAVJ genomes and chicken miRNAs (ver. 24.0) [55] using BLASTN-SHORT [62]. The parameters used for the analysis were extremely conservative (match/mismatch scoring set to 1/−3, gap open/extend penalty set to 3/3, and E-value set to 100) to identify homologies with 99% conservation [57]. Virus sequences were set as queries and miRNAs were used as subjects.

Highest scoring pairs (HSPs) where the viral sequence was identical to the chicken miRNA seed site in the same (5′-3′) orientation were considered vSMs (Supplementary File Table S1—vSMs). In contrast, if the match between the viral sequence and the miRNA seed site was in the anti-sense orientation, these HSPs were designated as vSSs (Supplementary File—vSSs). Hits that were outside the seed site were not considered for further analysis.

**Figure 1:**
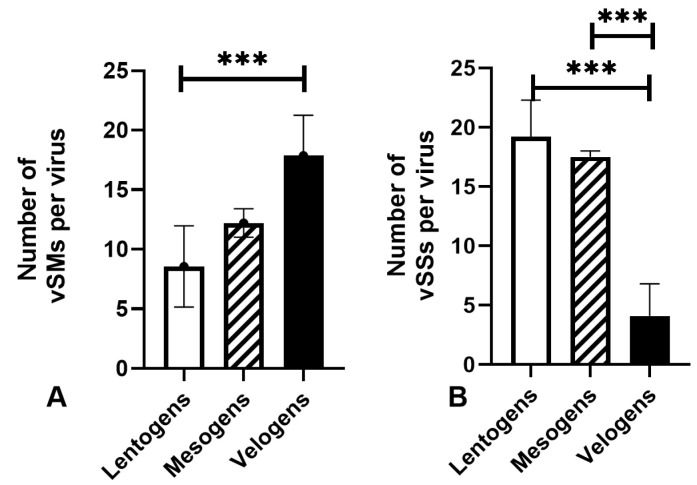
Differential abundance of viral seed mimics and seed sponges in AOAV-1 pathotypes. (**A**) Average number of viral seed mimics (vSMs) in lentogenic, mesogenic, and velogenic viruses are shown in clear, hatched, and filled bars, respectively. Data represent summary of Table 1 and Table 2 (*** indicates *p* < 0.001). (**B**) Average number of viral seed sponges (vSSs) in lentogenic, mesogenic, and velogenic sequences are shown in clear, hatched, and filled bars, respectively. Data represent summary of Table 1 and Table 2. Comparisons were completed using ordinary one-way ANOVA with Tukey’s multiple comparisons test with a single pooled variance.

The query and analysis identified 137 vSMs and 146 vSSs among the coding regions of 16 lentogenic strains; 61 vSMs and 33 vSSs in the coding regions of 5 mesogenic strains; and 1394 vSM and 255 vSSs in the coding regions of 78 velogenic strain sequences analyzed. The average number of vSMs was 8.5 for lentogenic strains, 15.5 for mesogenic strains, and 19.5 for velogenic strains, and this difference was statistically significant (*p*-value = 0.0008) between lentogens and velogens (Figure 1A). The mean number of vSSs per virus in lentogenic and mesogenic viruses was 19.2 vs. 17.5 and 4.09, respectively. This paucity of vSS sequences in velogens was statistically significant relative to both lentogens and mesogens (*p* < 0.0001) (Figure 1B).

To rule out that these were chance or spurious bindings between viral genomes and chicken miRNA seed sites, we scrambled the sequence of the OAVJ strain LaSota (accession: JF950510.1) twice sequentially using the sequence manipulation suite (SMS ver. 2.0) [63] without changing the overall sequence composition (46.2% G+C and 53.8% A+T of JF950510.1). The BLASTN homology search for both scrambled sequences did not pick up any sequences in the NR database, nor did they show any homology in a BLAST2sequences analysis with the OAVJ strain LaSota. A BLASTN-SHORT analysis of the scrambled LaSota sequence against the chicken miRNA database using the same parameters above failed to identify any vSMs or vSSs; however, one vSS match was identified between mir-27b-3p and the L gene in the OAVJ strain LaSota. This was a spurious seed match with a poor e-value (Supplementary Material—BLASTN-SHORT scrambled).

### 3.2. vSM and vSS Motifs Show Distinct Distribution Patterns

We next explored the distribution of vSMs and vSSs across the viral genomes to understand if these motifs were spread throughout or concentrated in certain genes or regions of the viral genomes. Counts of vSMs across genes were normalized to the number of sequences for each pathotype. The number of vSMs decreased in the following order: polymerase (L) > matrix (M) > fusion (F) > nucleoprotein (NP) > hemagglutinin (HN) > phosphoprotein (P) for lentogens; L > M > P > NP > HN > F for mesogens; and L > M > NP > P > F > HN for velogens (Table 1). L genes had an average of 7.8 and 8 vSMs in lentogens and mesogens, respectively, versus 9.0 vSMs in velogens. Velogens had significantly lower numbers of vSSs per virus relative to both lentogens and mesogens. The abundance of vSSs in lentogenic and mesogenic sequences decreased in the following order: L > F > intergenic regions > M > P > NP > HN (Table 1). The ratio of vSMs to vSSs across genes and pathotypes was different for the HN gene of mesogens and velogens; further, vSM/vSS ratios of M, NP, and L were higher than lentogens or mesogens. Similarly, although our initial hypotheses suggested that longer genes could potentially harbor more vSMs or vSSs, the ratio of vSMs or vSSs per gene kb did not vary substantially (Table 1). The analysis of vSM or vSS hits from intergenic regions did show higher numbers of vSM vs. vSS sequences, as observed with the coding regions; however, the vSM/vSS ratio was not statistically significantly different between lentogens and mesogens or velogens (Table 1 and Supplementary File—Intergenic_hits).

We also evaluated the presence and expression of miRNAs targeted by the vSMs and VSSs identified above in chicken tissue. The miRNA expression database for chicken tissues was downloaded from miRbase and mirGeneDB [64]; then, hits that could have an impact on viral replication and host gene expression were shortlisted by comparing for miRNAs that had ≥10 reads with the vSMs and vSSs identified above (Table 2).

**Table 1:** Distribution of vSM and vSS motifs in different OAVJ genes by pathotype. Total number of vSMs or vSSs per gene, numbers per pathotype, mean numbers per virus, and per kb gene length were calculated and tabulated as described in text. Average gene lengths were calculated from feature tables for each virus sequence using Geneious 9.2. N.A = not applicable.

				Per Sequence			Per kb Gene Length	
	vSM	vSS	Average Gene Length	vSM	vSS	vSM/vSS Ratio	vSM	vSS
**Fusion (total)**	120	79						
Lentogenic	16	30	1662	1.0	1.9	0.5	1.07	1.13
Mesogenic	5	2	1662	1.0	0.4	2.5	0.60	0.24
Velogenic	99	47	1662	1.3	0.6	2.1	0.76	0.36
**HN (total)**	88	20						
Lentogenic	14	10	1787	0.9	0.6	1.4	0.49	0.35
Mesogenic	5	1	1746	1.0	0.2	5.0	0.57	0.11
Velogenic	69	9	1721	0.9	0.1	7.7	0.51	0.07
**Matrix (total)**	281	64						
Lentogenic	19	20	1095	1.2	1.3	1.0	1.08	1.14
Mesogenic	7	3	1095	1.4	0.6	2.3	1.28	0.55
Velogenic	255	41	1095	3.3	0.5	6.2	2.99	0.48
**Nucleoprotein (total)**	212	45						
Lentogenic	15	19	1470	0.9	1.2	0.8	0.64	0.81
Mesogenic	5	5	1470	1.3	1.3	1.0	0.68	0.68
Velogenic	192	21	1470	2.5	0.3	9.1	1.67	0.18
**Phosphoprotein (total)**	125	58						
Lentogenic	3	13	1191.2	0.2	0.8	0.2	0.16	0.68
Mesogenic	7	7	1190	1.4	1.4	1.0	1.18	1.18
Velogenic	115	38	1188	1.5	0.5	3.0	1.24	0.41
**Polymerase (total)**	766	168						
Lentogenic	70	54	6615	4.4	3.4	1.3	0.66	0.51
Mesogenic	32	15	6615	6.4	3.0	2.1	0.97	0.45
Velogenic	664	99	6615	8.5	1.3	6.7	1.24	0.41
**Intergenic (total)**								
Lentogenic	5	2	N.A	0.3	0.1	3	N.A	N.A
Mesogenic	2	2	N.A	0.4	0.4	1	N.A	N.A
Velogenic	57	39	N.A	0.7	0.5	1.4	N.A	N.A

**Table 2:** Comparing predicted vSMs and vSSs to miRNA expression dataset to identify top candidates. vSM and vSS predictions were compared to miRNA expression datasets as indicated. Numbers in column two represent normalized read counts from [65]. NP = Nucleoprotein, P = Phosphoprotein, M = Matrix, HN = Hemagglutinin-Neuraminidase, F = Fusion, and L = Polymerase. Lento = lentogen, Meso = mesogen, Velo = velogen, In = intergenic. Numbers in parentheses indicate number of sequences in that pathotype. Candidates indicated with * were analyzed further. Expression data were obtained from mirgenedb.org. K = 1000 reads.

		miRNA Reads per Million	Target Gene	Pathotype Found in
**vSM**	gga-miR-148b-3p *	42–43.5K	L	Lento (1); Velo (1)
	gga-miR-15b-5p *	275	L	Velo (11)
	gga-miR-15c-5p	250	L	Velo (11)
	gga-miR-129-5p *	259	NP	Lento (1)
	gga-let-7a-2-3p	12.5K	M	Velo (2)
	gga-miR-12211-3p	21	L	Velo (55)
	gga-miR-1782	20	HN	Velo (1)
	gga-let-7a-3p	19	L	Velo (28)
	gga-let-7k-3p	5.0K	L	Velo (28)
	gga-miR-144-5p	75–90	L	Velo (1)
	gga-miR-1769-3p	0	L	Velo (1)
	gga-miR-12277-5p	0	L	Velo (1)
	gga-miR-1759-5p	0	L	Lento (1); Velo (8)
**vSS**	gga-miR-21-5p	13.0K	L	Lento (4); Meso (2)
	gga-miR-26a-2-5p	44.4–46.5K	L	Lento (7); Velo (14)
	gga-miR-101-3p	11.1K	L	Lento (1)
	gga-miR-30c-5p	6.0K	M	Meso (1), Velo (15)
	gga-miR-30d	~20K	M	Lento (1); Velo (14)
	gga-miR-26a-5p	46.5K	F	Lento (7); Velo (14)
	gga-miR-30a-5p	~35.0K	M	Lento (2); Meso (1); Velo (13)
	gga-miR-30b-5p	1.0K	M	Lento (8); Meso (1), Velo (2)
	gga-miR-30c-1-3p	6.0K	IN	Lento (13)
	gga-miR-27b-5p *	~4.5K	P	Lento (2); Meso (1); Velo (14)
	gga-miR-182-5p	~2.0K	M	Lento (4)
	gga-miR-30e-5p	2.5–17.5K	M	Lento (1)
	gga-miR-144-5p	75–90	L	Velo (4)
	gga-miR-1769-3p	0	L	Lento (8)

### 3.3. Sequences Flanking vSMs Show Characteristic Stem–Loop Folding Patterns

**Figure 2:**
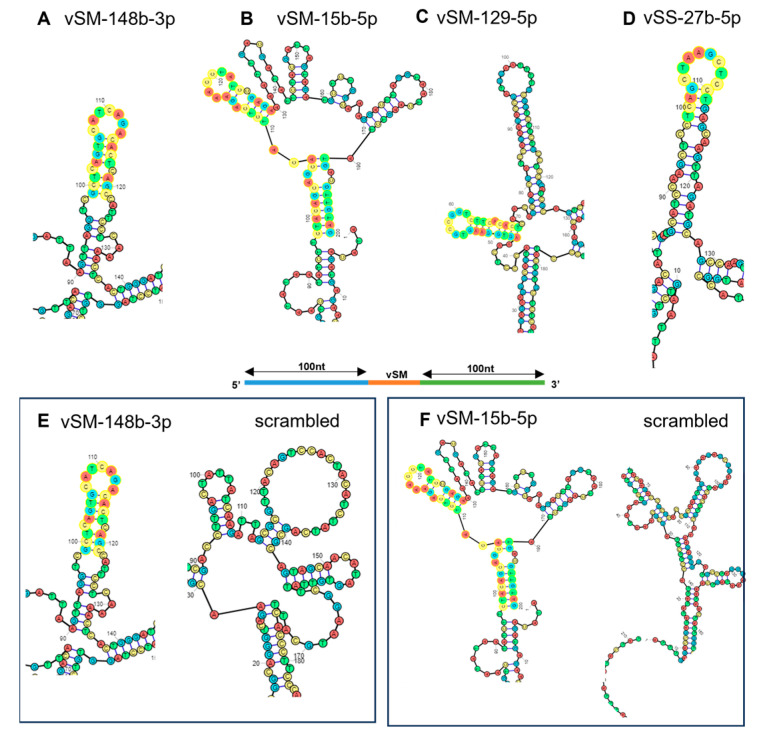
RNA secondary structure analysis identifies prominent stem–loop structures in vSMs and vSSs of interest. Secondary structure predictions of 100 nucleotides flanking vSM-148b-3p (**A**), vSM-15b-5p (**B**), vSM-129-5p (**C**), and vSS-27b-5p (**D**) are shown. The most stable structure based on lowest mean free energy, as predicted by RNAstructure with default parameters, is shown. Ct files obtained from RNAstructure were then analyzed using Ribosketch [69]. vSM and vSS residues are highlighted. Effects of shuffling the sequences of vSM-148b-5p (**E**) and vSM-15b-5p (**F**) are shown. Left panel represents secondary structure of original vSM, while the right panel represents the secondary structure of the scrambled sequence. Highlighted nucleotides (beginning at position 101) show the predicted vSM/vSS sequence. Only relevant parts of the full structure are shown for clarity. For vSS-27-b-5p, the reverse complement of the sequence is shown for clarity.

A characteristic feature of miRNA precursor sequences is the ability to form stem–loop structures [66]. We hypothesized that vSMs mimic host miRNA function. To investigate if the vSM and vSS candidates identified here could be targets for RNAse III cleavage, we extracted 100bp regions around the predicted vSMs or vSSs from corresponding viral genomes and then analyzed their secondary structure using RNAstructure [66]. For the top three vSM candidates in Table 2 (vSM-148b-3p, vSM-15b-5p, and vSM-129-5p) and vSS-27b-5p, the RNA secondary structure prediction showed a very stable stem–loop structure similar to the miRNA precursor sequences (Figure 2). MiRNA precursor sequences exhibit these stem–loop conformations and are processed into the mature miRNA duplex by class III RNase enzymes involved in miRNA biogenesis [67,68]. Thus, viral sequences that fold to similar structures may be templates for these enzymes and generate small RNAs from viral transcripts capable of mimicking host miRNA seed sequences. To ensure that these structures were predicted only when the vSM was present, vSM-148b-3p, vSM-15b-5p, and vSM-129-5p sequences were randomized in two independent rounds, keeping the sequence composition (A+T/G+C) the same; all homology to the corresponding miRNAs, as measured using BLAST2sequences, was lost. Scrambling vSM-148b-3p led to shorter stems (5 bp stems) and a decrease in the mean free energy for the predicted structure (Figure 2E,F). Similar data were observed for scrambled miR-15b-5p and scrambled miR-129-5p. These data suggest that these regions in the viral genome can be organized into stem–loop structures that can be templates for miRNA processing machinery.

### 3.4. Sequence Complementarity Determines Function

**Figure 3:**
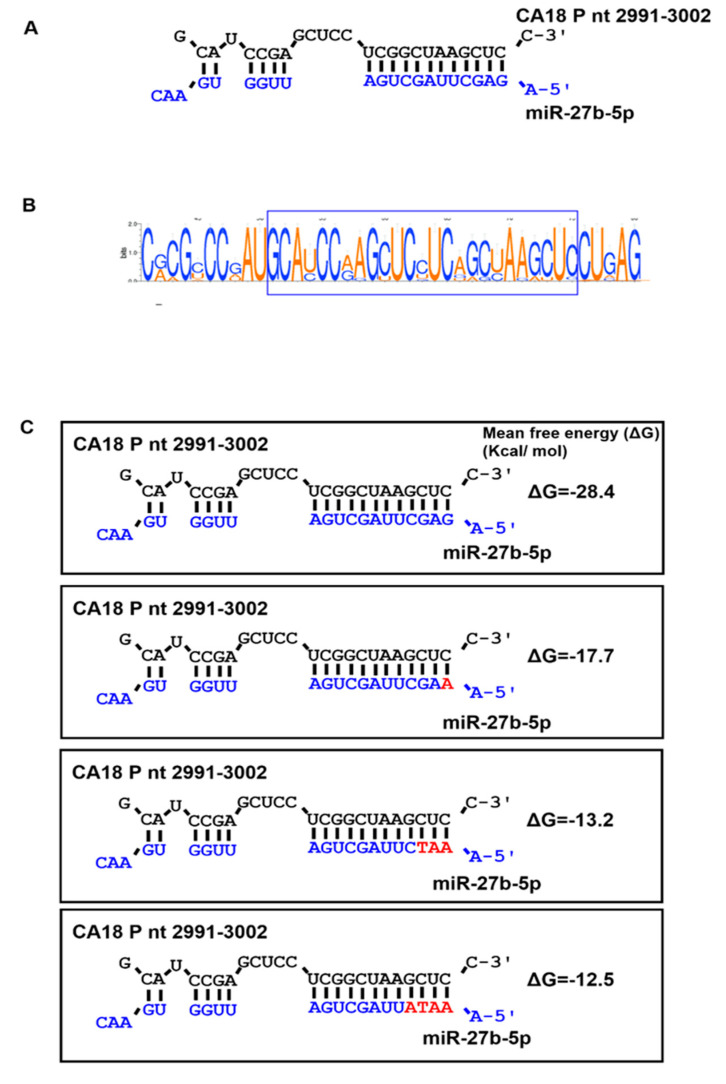
Analysis of vSS-miR-27b-5p: (**A**) Sequence alignment of OAVJ P 2991-3002 interaction with gga-miR-27b-5p, as predicted by RNAhybrid, is shown. Straight lines indicate Watson–Crick base pairing. (**B**) Sequence logo demonstrates conservation of vSS-miR-27b-5p in all OAVJ strains analyzed. Height of letters corresponds to conservation. The vSS region is boxed. (**C**) Predicted mean free energy for miR-27b-5p-P gene transcript calculated from RNAhybrid and effect of progressive in silico point mutation on thermodynamic stability of the heterodimer are shown. Black sequence represents OAVJ P gene transcript while dark-blue sequence represents gga-miR-27b-5p. Changes to seed sequences are shown in red.

Among the key vSSs identified in this analysis, we shortlisted vSS-27b-5p for experimental validation for multiple reasons: (1) vSS-27b-5p is encoded in the OAVJ P gene between nucleotides 2991 and 3002; (2) interaction between vSS-27b-5p and its target miRNA miR-27b-5p was predicted to be thermodynamically stable by RNAhybrid [70] (Figure 3) with a mean free energy of −28.4 kcal/mol; (3) the alignment of nucleotides 2900–3100 in the P gene locus and sequence logo visualization [71] showed a high degree of conservation (Figure 3B); and (4) alternative transcripts of the P gene (V and W) have been shown to dampen the host interferon response [72,73]. Scrambling the P gene sequence while maintaining sequence composition resulted in loss of complementarity between miR-27b-5p and the P gene transcript, which highlights the binding specificity of the miRNA and the gene. Similarly, the in silico replacement of a single nucleotide in the miR-27b-5p seed site from G to A reduced the predicted thermodynamic stability of the complex with the OAVJ P gene from −28.4 to −17.7, with a progressive decrease in stability down to −12.5 with the modification of four nucleotides (Figure 3C). Overall, the duplex was predicted to be thermodynamically more unstable as one or more changes to the seed site were made.

**Figure 4:**
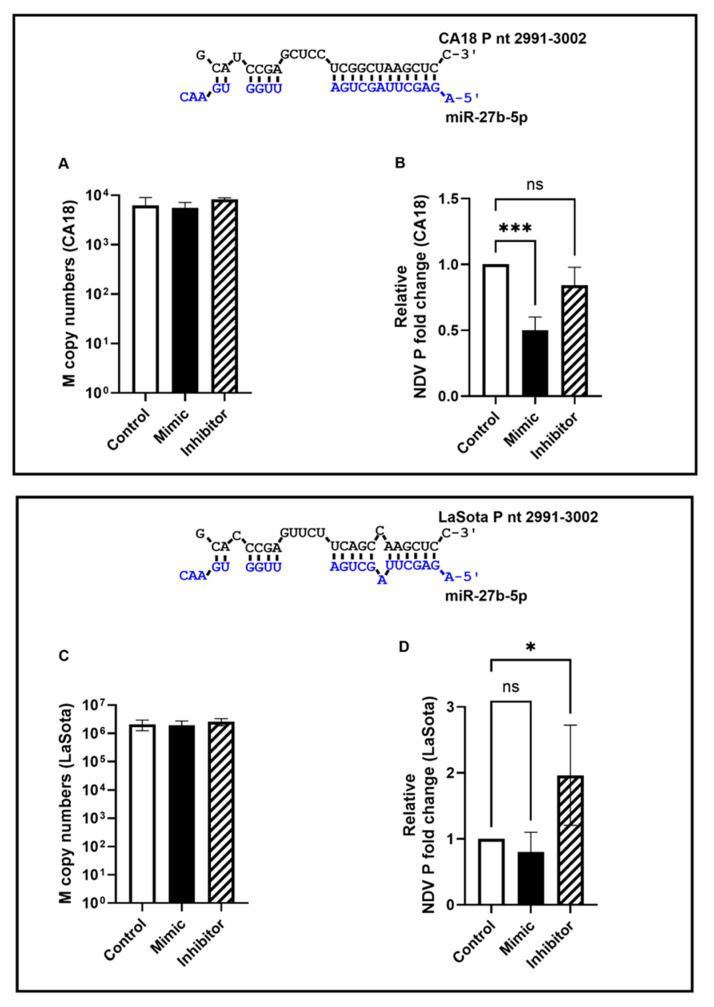
vSS-miR-27b-5p modulates expression of CA18 M and P genes. Total RNA from chicken fibroblast (DF-1) cells infected with CA18 followed by transfection with controls or miR-27b-5p mimics/inhibitors was analyzed for expression of OAVJ M gene copy numbers (**A**,**C**) or fold change in P gene relative to glyceraldehyde phosphate dehydrogenase (GAPDH) (**B**,**D**). Panels A and B represent infection with CA18, while panels C and D represent infection with OAVJ strain LaSota. Data represent mean ± SD from triplicates. Standard curves conform to MIQE guidelines. ns = non-significant; * *p* < 0.05, *** *p* < 0.0005. Statistical significance was calculated using one-way ANOVA with single pooled variance and post hoc Dunnett’s test.

Since the majority of vSS-27b-5p matches were identified in velogenic strains, we next tested the effects of miR-27b-5p upregulation or inhibition on viral replication and P gene transcript levels using the velogenic CA18 outbreak strain of OAVJ. Chicken fibroblast (DF-1) cells were first infected with CA18 (MOI = 5.0) for 1 h and then transfected with the miR-27b-5p mimic, inhibitor, or controls to determine the direct effect of miR-27b-5p on viral replication, if any. Copy numbers of the M gene (as a measure of viral replication) and P gene (a predicted target for miR-27b-5p) were determined at 24 h post-infection. The levels of M gene between the control and mimic-treated cells were equivalent, whereas a small (but statistically not significant) increase in M copy numbers was observed in inhibitor-treated cells (Figure 4A). MiR-27b-5p overexpression led to a statistically significant decrease in P gene transcripts relative to the controls compared to the inhibitor treatment (Figure 4B), suggesting that for CA18, miR-27b-5p overexpression led to the degradation of P gene transcripts, which was not observed when miR-27b-5p binding was inhibited. It is important to note that, in the case of CA18, there is perfect complementarity between the P gene transcript and miR-27b-5p over the entire length of the miRNA seed site.

In contrast, perfect complementarity was not observed between the miR-27b-5p seed site and the P gene transcript of the OAVJ LaSota strain (specifically, the U residue at nucleotide 8). Transfection of the miR-27b-5p mimic did not alter M gene expression (Figure 4C) but led to a small but statistically insignificant decrease in the expression of the P gene relative to the control transfected cells (Figure 4D). A small but statistically significant increase in P gene transcripts was observed in inhibitor-transfected cells (Figure 4D). These data show that, owing to a lack of complementarity over the entire seed region, miR-27b-5p is likely unable to bind as strongly to LaSota P transcripts. Overall, these data show that vSS-miR-27b-5p in P gene transcripts can bind to chicken miR-27b-5p during infection, and the alteration of miRNA levels can modulate P gene expression.

## 4. Discussion

After infection with OAVJ, viral transcripts are known to modify the host immune response and cell cycle to facilitate replication [47,48,49]. OAVJ gene transcription follows the prototypical paramyxovirus model, in which genes at the 3′ end of the genome are transcribed at a much higher level compared to those at the 5′ end due to polymerase slipping. The error-prone nature of viral RNA-dependent RNA polymerase (L) introduces mutations in viral transcripts, leading to a viral quasispecies [74,75] with mutation rates of up to 1 for every 10,000 bases in the case of influenza viruses [76]. It is also known that viral transcripts can constitute a large fraction of the total RNA in a cell during infection [77]; it is likely that nonsense viral transcripts produced during OAVJ infection and error-prone replication may modify the function of chicken miRNA networks to promote viral replication and/or persistence, and alter how viral transcripts interact with host RNAs.

In this article, we provide in silico analyses showing that OAVJ genomes potentially encode viral miRNA seed mimics (vSMs) and sponges (vSSs) (Figure 1). vSMs were discovered in all three pathotypes; however, the number of vSMs per virus genome were significantly higher in velogenic strains compared to lentogenic or mesogenic strains (Figure 1). Using a stringent filtering criterion, we also identified vSS motifs in lentogenic and mesogenic viruses, but not in velogenic viruses. The density of vSM motifs was not related to gene length, as all pathotypes had a roughly equivalent ratio of vSMs in all six genes (Table 1).

The analysis of one conserved vSS (vSS-miR-27b-5p) identified in the P gene was predicted to have a thermodynamically stable interaction with the target miRNA, and this signature was found to be conserved (Figure 3) among velogenic strains especially. Altering a few residues in the seed site modified the predicted thermodynamic stability of the miR-27b-5p and OAVJ P interaction, suggesting that the seed site is likely important for this interaction. The transfection of chicken fibroblast cells with miR-27b-5p inhibitors and mimics modulated levels of P transcripts only for the CA18 outbreak strain of OAVJ, but not the LaSota strain.

The small increase in LaSota P gene transcripts in miR-27b-5p inhibitor-transfected cells suggests that alternative cellular pathways are likely involved as well, but this was not explored in this study. This needs further investigation using inducible wild-type or site-directed mutant P gene overexpression systems and/or reporter assays using such systems. This is indeed likely since mimic transfections can upregulate endogenous miRNA levels to non-physiological levels (personal observations). Evidence in other vertebrate species suggests that miR-27b has a potent anti-viral function in the case of mouse cytomegalovirus (MCMV) [66], transmissible gastroenteritis virus (TGEV) [78], and hepatitis C virus [79]. Importantly, miR-27b transcripts are cleaved by viruses such as MCMV [66]. In the context of poultry, miR-27b is upregulated during infection with Infectious Bursal disease virus (IBDV) and suppresses viral replication, upregulating the expression of ISGs like interferon beta (IFN-β), interferon regulatory factor 3 (IRF-3), and nuclear factor kappa beta (NF-κβ) [80,81]. Influenza A infection in chicken trachea has been shown to induce miR-27b expression and postulated to regulate the expression of tumor growth factor beta (TGF-β), mitogen-activated kinase (MAPK), Toll-like receptors (TLRs), and suppressors of cytokine signaling 6 (SOCS-6) [82]. In other systems, miR-27b has been demonstrated to regulate multiple cellular genes, such as carbonic anhydrase X (CA10) [83], as well as transcription factors such as c-Jun, peroxisome proliferator-activated receptor alpha (PPARα), and hepatocyte nuclear factor 4 alpha (HNF4α) [84]. The in silico prediction of the miRNA structures (Figure 2) suggests that there are stem–loop structures which may be targeted for cleavage by class III RNases, suggesting a possible mechanism for OAVJ-encoded miRNAs similar to those found in MCMV.

These data suggest that RNA-RNA interactions between host and viral transcripts can modify the outcomes of infection. RNA viruses have been shown to interact with host miRNAs directly or indirectly and modulate viral replication [85,86,87]. The data in this manuscript do not imply that OAVJ transcripts encode miRNAs, but rather suggest that viral transcripts may be able to mimic or inhibit host miRNA activity via seed site mimicry or sequestration. While this study focused on viral–miRNA interactions, other RNA-RNA interactions are not uncommon. The abundance of vSMs in velogens suggests that velogenic OAVJs may potentially be able to repress host gene expression to a much greater extent relative to lentogenic/mesogenic viruses. This is indirectly supported by previous observations that (a) velogenic infections in chickens are typically systemic, acute, and induce significantly higher mortality, which stems from an exacerbated immune response, as indicated by different cytokine expression profiles [88]; (b) microRNAs have been documented to regulate both pattern recognition receptors and interferon and cytokine expression during viral infections, either directly or indirectly via the regulation of secondary regulators [89,90,91,92]; and (c) miRNAs and their target genes or pathways tend to be conserved. Thus, vSMs in viral transcripts may be able to subvert the regulation of the innate and adaptive immune response during infection, and the higher number of vSMs confers velogenic viruses with an evolutionary advantage to not only infect, but also spread intra- and interspecies. Further analysis of historical OAVJ viruses and sequences to determine the diversity of vSM and vSS signatures may improve our understanding of how velogenic viruses accumulate these vSM signatures and potentially evolve in fitness. The expression of many host genes is regulated by competitive endogenous RNAs (ceRNA) [21,93,94,95,96,97]. Many host miRNAs can directly target viral transcripts and downregulate their translation [98,99,100,101]. Following OAVJ infection, the gradient transcription of viral genes may generate transcripts that can either mimic host miRNAs or sequester miRNA activity. These vSS and vSM sequences may deregulate regular host gene pathways (cell cycle [102], innate immune sensing, etc.) and potentially facilitate the replication of OAVJ. OAVJ-encoded vSSs may block the normal miRNA inhibition of pro-viral genes, increase pro-viral protein levels, and allow increased viral replication; this is especially true for vSMs that are unique to velogenic strains. vSS sequences may also block the binding of viral miRNAs to viral transcripts, maintaining viral protein levels and viral replication. OAVJ vSMs could mimic host miRNA activity, directly target host anti-viral genes, and modify viral replication.

The information gleaned from these RNA-RNA interactions improves our understanding of host–virus biology and may aid in improving current assays and developing new methodologies to differentiate velogenic from lentogenic viruses. Advancing knowledge of the host immune response to viral infection and the role of viral miRNAs in immune mechanisms could be useful for future antiviral and vaccine development.

## Data Availability

All data for this manuscript are included in the Supplementary Material and are available from the corresponding author upon reasonable request.

## Supplementary Materials

The following supporting information can be downloaded at: https://www.mdpi.com/article/10.3390/v16111748/s1.
